# A data-driven individual-based model of infectious disease in livestock operation: A validation study for paratuberculosis

**DOI:** 10.1371/journal.pone.0203177

**Published:** 2018-12-14

**Authors:** Mohammad A. Al-Mamun, Rebecca L. Smith, Annette Nigsch, Ynte H. Schukken, Yrjo T. Gröhn

**Affiliations:** 1 Department of Epidemiology of Microbial Diseases, Yale School of Public Health, New Haven, Connecticut, United States of America; 2 Department of Pathobiology, University of Illinois, College of Veterinary Medicine, Urbana, Illinois, United States of America; 3 Department of Animal Sciences, Wageningen University, Wageningen, The Netherlands; 4 Department of Population Medicine and Diagnostic Sciences, Cornell University, College of Veterinary Medicine, Tower Road, Ithaca, New York, United States of America; University of Illinois, UNITED STATES

## Abstract

Chronic livestock diseases cause large financial loss and affect animal health and welfare. Controlling these diseases mostly requires precise information on both individual animal and population dynamics to inform the farmer’s decisions, but even successful control programmes do by no means assure elimination. Mathematical models provide opportunities to test different control and elimination options rather than implementing them in real herds, but these models require robust parameter estimation and validation. Fitting these models to data is a difficult task due to heterogeneities in livestock processes. In this paper, we develop an infectious disease modeling framework for a livestock disease (paratuberculosis) that is caused by *Mycobacterium avium* subsp. *paratuberculosis* (MAP). Infection with MAP leads to reduced milk production, pregnancy rates, and slaughter value and increased culling rates in cattle and causes significant economic losses to the dairy industry. These economic effects are particularly important motivations in the control and elimination of MAP. In this framework, an individual-based model (IBM) of a dairy herd was built and MAP infection dynamics was integrated. Once the model produced realistic dynamics of MAP infection, we implemented an evaluation method by fitting it to data from three dairy herds from the Northeast region of the US. The model fitting exercises used least-squares and parameter space searching methods to obtain the best-fitted values of selected parameters. The best set of parameters were used to model the effect of interventions. The results show that the presented model can complement real herd statistics where the intervention strategies suggest a reduction in MAP prevalence without elimination. Overall, this research not only provides a complete model for MAP infection dynamics in a dairy herd but also offers a method for estimating parameters by fitting IBM models.

## Introduction

Chronic livestock diseases like paratuberculosis (PTB) and bovine tuberculosis (bTB) are commonly reported worldwide [1,2]. Bovine TB is caused by the pathogen Mycobacterium bovis (*M*. *bovis*) while PTB is caused by *Mycobacterium avium* subsp. *paratuberculosis* (MAP). In the UK, bTB has been spreading over the last two decades, putatively due to the presence of a wildlife reservoir in badgers [3]. In the United States (US), 68% of dairy herds have apparently at least one cow that is infected with MAP [4]. Both diseases pose a potential threat not only to animal health and production, but also to public health. Historically, bTB has been a contributor to human TB cases worldwide and PTB infections in humans have been associated with an increased risk of Crohn’s disease in humans [5]. Recently, it has been reported that these diseases may pose additional collateral risks for public health due to dispensed antibiotics as a treatment in some cases contributing to the spread of antibiotic resistance [6].

In the US cattle industry, the cost of PTB was estimated at $250 million every year [7]. The MAP Infection usually occurs in the first year of a ruminant’s life [8] and transmission can occur vertically [9] and/or horizontally via ingestion of fecal material contaminated by MAP [10]. As PTB is a slowly progressive disease, the progression of individual animals through different MAP infection states is a complex continuous process alternating excreting and non-excreting stages with a late onset of clinical signs [11,12]. It has a large economic impact for producers due to decreased milk production [13–15], premature culling [16,17], reduced slaughter value [18], low fertility [19,20], and an increased animal replacement rate [21]. However, MAP is difficult to diagnose due to the long incubation period [22,23], lack of early clinical signs [24–26], and imperfect testing [27].

In the last two decades, different mathematical models have been developed on a within-herd scale to understand MAP transmission dynamics [28,29] and effectiveness of recommended control strategies [30–33]. These models were used to assess the impact of contact structure on the MAP transmission [28], efficacy of test-and-cull policy [29,30,34,35], impact of low diagnostic test sensitivity in decision making [8,36], stopping some transmission pathways using hygiene improvement [37], improved calf management [38], impact of super-shedders in transmission [25,39], and economic efficacy of recommended programs [34]. Most of these studies suggest that culling a test positive animal is an effective solution to reduce the prevalence within a herd. However, none of the previous models considered the pervasiveness of MAP in the farm environment and the value of individual animal information along with real dairy herd data. Moreover, controlling MAP requires management of testing and culling strategies to reduce the prevalence, but these are unregulated and reliant on farmers’ decisions [40]. The decision to cull an animal is not straightforward and poses a multiscale problem where an individual animal, farm dynamics, infectious status, disease symptoms, and management profit are related [41]. Substantial costs are also related to the implementation of control measures and prevention [21,25,42]. Previous MAP models have explored many potential interventions programs, most considered population-level decision making rather than individual-level animal information. Recently, individual-based models (IBMs) have been proposed to show the value of the information about the infection, daily life events and management policy for each individual animal within the farm [37,41,43–46].

Mathematical models of infectious diseases are tools to enhance the understanding both of infection biology and efficacy of intervention policies in human and veterinary medicine [47]. However, translating modeling results into practice requires appropriate real-world assumptions to be built into the model. We hypothesize that in case of MAP, use of model results will be more realistic when the model has been built on up-to-date infection biology and epidemiology, parametrized from adequate real herd data, and fitted back to that real-world scenario to test the recommended intervention strategies. In this paper, our aim is to build an IBM framework of MAP infection that is fitted to and validated by in-depth longitudinal data from three northeastern dairy farms. The objective of this study was four-fold: first, we extended an existing IBM of a dairy herd to resemble the population level parameters (i.e. milk yield, herd size) with three real herds to create three *in silico* herds; second, we fitted the milk-yield measurement of individual animal to those herds; third, we fitted the model-predicted apparent MAP prevalence to the observed data to obtain herd-specific infection parameters; and fourth, we integrated a risk-based control strategies on those three *in silico* herds to evaluate the efficacy of risk-based controls. Finally, we discuss the value of observational data to feed information to simulation models, thereby making simulations more reflective and predictive of real-world circumstances.

## Materials and method

### The Individual-based model

We used a multiscale agent-based simulation of a dairy herd (MABSDairy), an improved version of dairy herd published in Al-Mamun et al. [37,44]. The MABSDairy is a multiscale stochastic IBM that simulates individual cows in a standard US cattle herd with a daily time step. In brief, each cow resides in one of three different management operations: adult/milking (aged >720 days), calf (aged 1–60 days) and heifer rearing housing (aged 61–719 days). Adult cows must calve to produce milk and the lactation cycle refers to the period between one calving and the next. The lactation cycle includes the processes of a voluntary waiting period (interval during the postpartum period), insemination, and the dry off period (a non-lactating period prior to an impending parturition to optimize milk production in the subsequent lactation). For fitting purpose, we modified the milk production Wood lactation curve by adding a herd-specific term and a herd-specific random component [48]. The function is defined as

$$Y_{t}\;=\;ad^{b}e^{ct}+f_{i}*f_{r}$$

wherei=Parity1and2byfarmA,B,andC

where *Y*_*t*_ is the yield on day t after calving, d is days in milk (DIM), *a* is a scaling factor for initial yield, *b* is a rate factor for the increase in yield to peak, *c* is a rate factor for the decline after the peak, f_i_ farm specific factor and f_r_ is a random number. We used base milk yield parameters from Dematawewa et al. for parities 1 and ≥2 in the basic model [49].

### MAP infection dynamics

**Fig 1:**
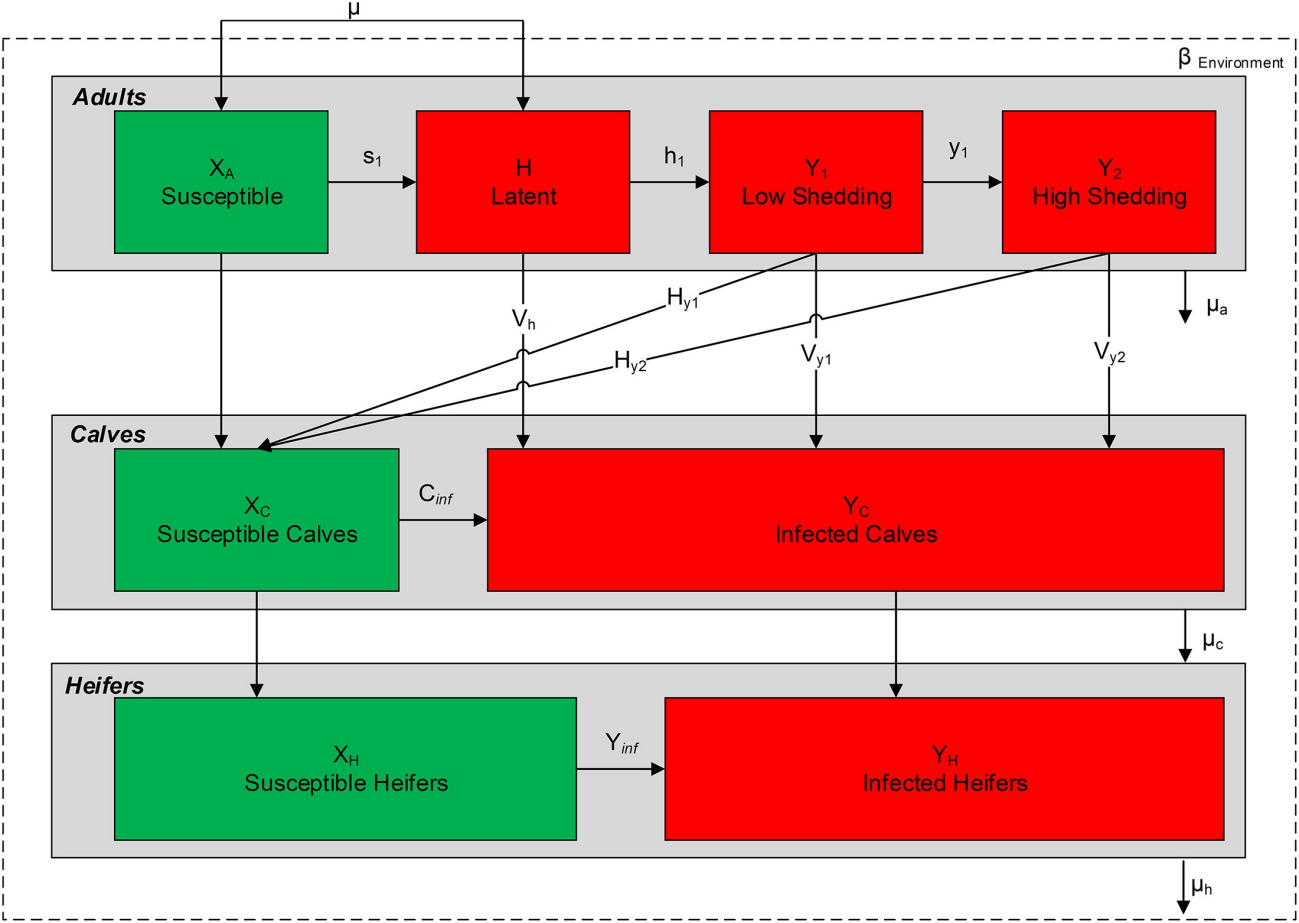
A flow diagram of animal movements among infection categories for the adult, calves, and heifers within the herd. Each horizontal gray box classifies the animals according to their initial age group. The green and red boxes define the susceptible and infected states, respectively, for each animal in the three age categories. The probabilities of exit at each time point from susceptible to latent, latent to low shedding and low shedding to high shedding animals are s_1_, h_1_, and y_1_, respectively. Vertical transmission probabilities from latent, low shedding and high shedding animals are V_h_, V_y1_, and V_y2_, respectively. Horizontal transmission probabilities to calves from low shedding and high shedding animals are H_y1_ and H_y2_, respectively. The probability an animal gets infected by the environment is *β*_*environment*_. Calf-to-calf and heifer-to-heifer transmission probabilities are C_*inf*_ and *Y*_*inf*_, respectively. Stochastic death/sale probabilities for adult, calves, and heifers are μ_a_, μ_c,_ and μ_h,_ respectively. *μ* is the replacement animals coming from heifer compartment upon completion of two years.

The infection compartments in the milking herd were divided into four categories: susceptible (X_A_), latent (H), low shedding (Y_1_), and high shedding (Y_2_). In calf rearing housing, there were two infection categories: susceptible (X_C_) and infected (Y_C_). In heifer rearing housing, there were also two infection categories: susceptible (X_H_) and infected (Y_H_). We included six different transmission routes: adult-to-adult, adult-to-calf (vertical transmission), adult-to-calf (horizontal transmission), environmental contamination, calf-to-calf, and heifer-to-heifer. The detailed infection structure is shown in Fig 1.

In the milking herd group, adult animals could be infected by low and high shedding adults. The probability of fecal-oral transmission for adult animals can be given by:

$${Inf}_{adult-adult}\;=\;\beta_{A}\left(\frac{\left(\beta_{direct}+\beta_{environment}\right)}{N}\right)$$

$$\beta_{direct}\;=\;\beta_{Y_{1}}Y_{1}+\beta_{Y_{2}}Y_{1}$$

$$\beta_{environment}\;=\;U\;(0,1)$$

Susceptible adult animals in the milking herd compartment were susceptible to MAP infection by contact with low shedding (*Y*_*1*_) and high shedding (*Y*_*2*_) animals with transmission rates of $\beta_{Y_{1}}$ and $\beta_{Y_{2}}$, respectively. β_A_ is the adult-to-adult transmission coefficient, *β*_*environment*_ is the MAP contamination risk from the environment and *N* is the total number of animals in the milking herd, *N = X*_*A*_*+H+Y*_*1*_*+Y*_*2*_. The horizontal infection probability to calves can be determined by

$${Inf}_{adult-calf}\;=\;\beta_{a}\left(\frac{{\beta_{direct}+\beta }_{environment}}{N_{c}}\right)$$

*β*_*a*_ is the horizontal transmission coefficient for an adult to newborn calves and N_c_ is the total number of calves at every day, N_c_ = Xc + Y_c_. A calf can also become infected vertically (i.e., in utero infection) by an adult and it is modelled using the certain proportions [30].

A calf stays in calf rearing housing for the first 60 days after birth. The probability of direct transmission was calculated as

$${Inf}_{calf-calf}\;=\;\alpha +\beta_{c}\left(\frac{{Y_{c}+\beta }_{environment}}{N_{c}}\right)$$

*β*_*c*_ is the horizontal calf-to-calf transmission coefficient, N_c_ is the total number of calves at each day, *X*_*c*_ is susceptible calves, *Y*_*c*_ is infected calves. During the first day after birth, a calf may also be infected horizontally by infected adults present in the maternity pen or vertically by an infected dam.

Susceptible calves became susceptible heifers and infected calves became infected heifers. Infected heifers could infect susceptible heifers by the heifer-to-heifer transmission path

$${Inf}_{heifer-heifer}\;=\;\beta_{h}\left(\frac{{Y_{h}+\beta }_{environment}}{N_{X_{H}}}\right)$$

*β*_*h*_ is the horizontal heifer-to-heifer transmission coefficient, and the total number of heifers is $N_{X_{H}}\;=\;X_{H}+Y_{H}$. After one year, the infected heifers became latent heifers and eventually entered the milking herd as latent adults. For simplifying the model, we assumed that heifer remains in the heifer rereading housing are transiently shedding while they ended up in the adult herd as latent animals.

### Observed herd data

The longitudinal dataset was obtained from a longitudinal study of three commercial dairy farms in the northeastern US: farm A in New York State, farm B in Pennsylvania, and farm C in Vermont [24,50]. All three farms participated in the Regional Dairy Quality Management Alliance (RDQMA) project, which was a multistate research program conducted under a cooperative research agreement between the USDA Agricultural Research Service, and four universities: Cornell University, Pennsylvania State University, University of Pennsylvania, and University of Vermont. The project consisted of longitudinal data collection for endemic infectious diseases of public and animal health concern in dairy herds. For a more complete description, including information on farms, samplings, and microbial analyses, see Pradhan et al. [50]. Briefly, the milking herds consisted of 330, 100, and 140 cows on farms A, B, and C, respectively. Sampling commenced in February, March, and November 2004 on farms A, B, and C, respectively, and continued for approximately 7 years, until 2010. The project design included a biannual collection of individual fecal samples and a quarterly collection of individual serum samples from all milking and non-lactating cows. Additionally, culled cows were tracked as much as possible from the farm to the slaughterhouse, where four gastrointestinal tissues and a fecal sample were collected with the cooperation of USDA Food Safety and Inspection Service personnel. The harvested tissues included two lymph nodes located at the ileocecal junction and two pieces of ileum, one taken from 20 cm proximal to the ileocecal valve and the other taken from very near the ileocecal valve. In addition to the sampling of animals, the farm environment was sampled in approximately 20 locations on a biannual basis. All fecal and environmental samples were tested by 4-tube culture for the presence of viable MAP organisms, reported as colony-forming units per tube. All serum samples were tested using the ParaCheck ELISA (Prionics USA Inc., La Vista, NE) for antibody reactions to MAP antigens. On each of the farms, demographic data, production data and herd management information were collected. Precise demographic data included birth date, birth location, calving dates, fertility data, animal location data (pen status at any point in time), dry-off dates, culling information and cull dates. These demographic data were collected for each animal present on the farms. All infection data, strain typing data, herd management, demographic, and production data were maintained in a relational database.

### Model parameters

The parameterization of the base dairy herd model is described in Al-Mamun et al. [37,51]. Initial infection parameter values were updated according to Mitchell et al. 2015 [47]. Table 1 provides the base parameters for the initial MAP transmission before fitting the model to the RDQMA herds.

**Table 1:** Base parameter values of *Mycobacterium avium* subsp. *paratuberculosis* (MAP) infection within a dairy herd.

Symbols	Description	Initial value	References
*V*_*h*_	The proportion of calves from latent animals infected at birth	0.15	[30]
*V*_*y1*_	The proportion of calves from low-shedding animals infected at birth	0.15	[30]
*V*_*y2*_	The proportion of calves from high-shedding animals infected at birth	0.17	[30]
*β*_*A*_	Adult-to-adult transmission coefficient	0.05	[37]
*β*_*a*_	Adult-to-calf transmission coefficient	0.383	[37]
*β*_*c*_	Calf-to-calf transmission coefficient	0.0025	[37]
*β*_*h*_	Heifer-to-heifer transmission coefficient	0.001	Calibrated in the model
$\beta_{y_{1}}$	Transmission rate between low shedders (Y_1_) and susceptible (X_A_)	2/year	Calibrated in the model
$\beta_{y_{2}}$	Transmission rate between high shedders (Y_2_) and susceptible (X_A_)	20/year	Calibrated in the model

### Model fitting method

The goal of the model-fitting exercise was to estimate key parameters in order to produce results consistent with the epidemiology observed on the three farms. Our fitting exercise was two-fold: first, we fitted our base dairy herd models with farm-specific parameters (total population and milk yield), then we fitted the model predicted apparent prevalence results based on antemortem ELISA and fecal testing and postmortem tissue and fecal testing results for the farm. To assess the goodness-of-fit we sampled from the defined parameter ranges in multiple rounds and ran the three in silico dairy herds. The model fitting was done using a nonlinear fitting method named Nelder-Mead Simplex Method [52], which is used for unconstrained optimization. While fitting the milk yield and apparent prevalence, the best-fit parameters were extracted.

To determine the specific range for each parameter, we used multidimensional parameter space searching method. The point estimate of each parameter was taken as a mean value and, using Latin Hypercube Sampling, 100,000 parameter combinations were generated spanning the specified range ±75% of the mean values. The searching was done in two stages. In the first stage, we set a broad range to identify the particular regions of the parameter range and chose the best 10,000 (10%) parameter sets. In the next stage, we ran the simulation with 10% parameter sets to compare with the best fit curve by minimizing the sum of square error. The parameter ranges presented in the results section were calculated from the top 1% simulations.

### Intervention strategies

Once the three *in silico* herds were stable using fitted values, we tested different proposed intervention strategies. We chose risk-based testing and culling strategies suggested by Al-Mamun et al. [37]. In brief, all cows that tested negative throughout testing were marked as low risk or green cows. The cows that tested positive were divided into two groups: yellow and red. Red animals had at least 2 positive tests out of the last 4 tests and yellow cows had one positive test. We proposed two controls: control I, culling red animals straightway (aggressive culling); and control II, culling only red animal with a delay of 305 DIM (delayed culling). The simulations results were then compared against the observed pre-fitted data from the three herds using three intervention strategies: no control, control I, control II.

### Simulation background

First, the base dairy herd model was initiated with a certain proportion of adult animals for farms A (330), B (100) and C (140). Second, after a 2 years burn-in period the model was run for 7 more years to resemble the observations of the real herds. During the 2 years burn-in period, each farm was assumed to be self-sufficient in producing their own replacement, so that no animal purchase from outside was needed. The model was initiated with a pre-determined distribution of animals with different parities. At each time step, the algorithm first determined the group of animals. If it found adult animals, it checked reproductive status (voluntary waiting period (VWP), waiting to be inseminated, and pregnant) and milk yield status. Any cow on the 280 days of pregnancy was assumed to calve. For a newborn calf, the stillbirth probability was checked; if the calf was not stillborn, it was flagged as a calf. Only female calves were kept in the herd, and male calves were removed immediately after birth. Once an adult animal calved, it transitioned to VWP status and continued in the milking herd loop until it was removed due to culling or death. Mortality was allowed in the calf rearing loop; otherwise, calves were transferred into the heifer loop at the 61st day of age. In the heifer loop, heifers were inseminated at the 400 days of age in order to become pregnant, so that they would calve at the 680 days of age. When heifers were ready to calve for the first time, they transitioned to the milking herd in the model. The model was fitted for the 7 years data for each farm. Third, for testing intervention strategies, each model was fitted to the first 4 years of data- that is called pre-intervention fit, and then the intervention was tested in 2 phases. In the first phase, 3 years and then extended more 2 years to see how the suggested strategies result in long-term. The base model was developed as custom codes in MATLAB and other data analysis were done using R.

## Results

The purpose of the fitting exercise was to obtain a better fit to the estimates of three herds prior fitting to the apparent prevalence. The model predicted total number of animals (adult, calves, and heifers) closely resembles the data from the three real farms (shown in Table 2).

**Table 2:** The comparison of observed and predicted values from three *in silico* farms in terms of a total number of animals, and average daily milk yield (in kg) for 305 days, presented as mean (95% confidence interval).

	Total number of animals	Milk yield: parity 1	Milk yield: parity ≥ 2
Herd A			
Observed	720 (708–754)	36.07 (29.61–40.73)	39.48 (27.11–49.86)
Predicted	714 (693–737)	36.15 (30.44–40.73)	39.49 (27.56–50.18)
Herd B			
Observed	194 (102–230)	33.38 (24.34–40.35)	34.52 (17.42–47.40)
Predicted	200 (182–219)	32.97 (26.51–38.31)	34.97 (21.86–48.29)
Herd C			
Observed	262 (116–339)	27.49 (19.03–34.68)	27.49 (19.03–34.68)
Predicted	221 (184–257)	27.16 (20.23–32.98)	27.90 (17.12–38.06)

**Fig 2:**
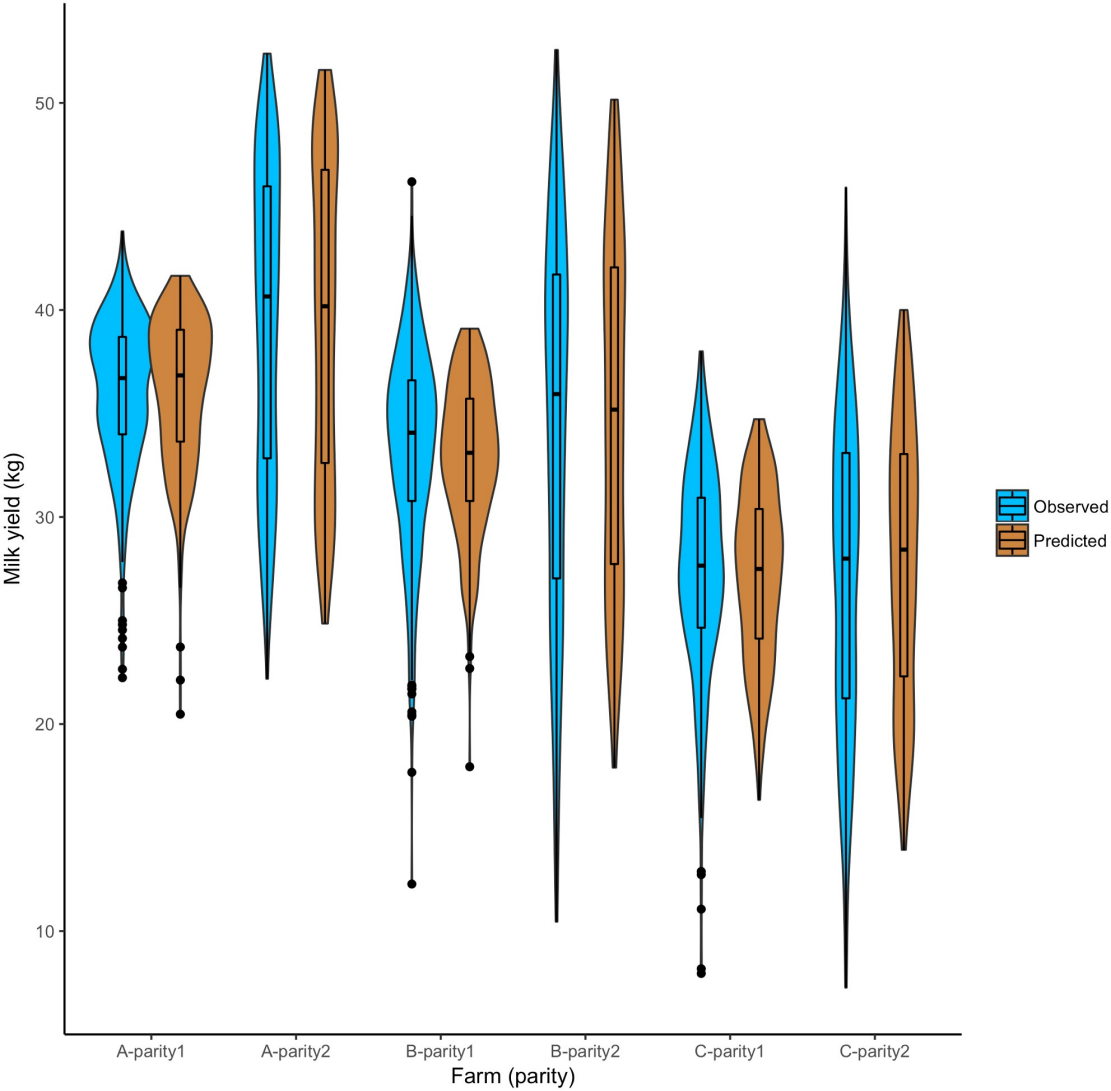
The comparison of observed and model predicted milk yield distribution for 1% simulation using best-fit parameters for the milk yield. In the box plot, the bottom and top end of the bars are minimum and maximum values respectively, the top of the box is the 75th percentile, the bottom of the box is the 25th percentile, and the horizontal line within the box is median; outliers are presented as a solid black circle and the density of the milk yield is presented by the width of the violin.

Fig 2 shows the concordance between predicted and observed milk yield data from three herds. It is evident that the models predicted milk yield estimations matched with the observed milk yield from three northeastern herds. The best fit model predictions to the observed milk yield curve for parity 1 and parity ≥2 are shown in supplementary S1 Fig. The best fitting lines also describe that the model was able to capture inherent randomness from the data into the model. The estimation of the critical parameters *a*, *b*, *c*, and *f*_*i*_ of the modified lactation curve are presented in Table 3.

**Table 3:** The estimated parameters from the fitting exercise for the modified milk yield function for three farms A, B, and C.

	*a*	*b*	*c*	Herd specific parameter (*f*_*i*_)
Farm A-Parity 1	17.87	0.21	0.0020	3.59
Farm A-Parity≥2	25.23	0.20	0.0033	5.19
Farm B-Parity 1	16.09	0.19	0.0020	6.60
Farm B-Parity≥2	23.38	0.20	0.0039	8.33
Farm C-Parity 1	15.25	0.19	0.0027	6.55
Farm C-Parity≥2	16.50	0.22	0.0040	9.00

### Model fitting exercise for disease parameters

Table 4 represents the observed apparent prevalence and apparent incidence and the tracking of the animals in the next biannual testing for three farms for seven years, 2004–2010. The observed prevalence shows zero infected animals in the last half of 2010, for the sake of persistence scenario we replace that with the previous quarter value. During our simulation, we normalized the prevalence with the previous half of the year so that it remains consistent for our simulation. We simulated the three *in silico* farms to fit with the observed apparent prevalence data from herd A, B, and C.

**Table 4:** The calculation of apparent prevalence and apparent incidence and the tracking of the animals in the next testing in bi-annually phase for three farms (2004–2010).

Year	2004		2005		2006		2007		2008		2009		2010	
Test phase	1	2	3	4	5	6	7	8	9	10	11	12	13	14
**Herd A**														
Total positive cows ^a^	14	25	34	28	34	32	21	23	24	17	14	9	7	0
Animals tested	315	330	364	349	354	364	338	332	337	347	341	347	296	239
Apparent prevalence	4.4	7.6	9.3	8.0	9.6	8.8	6.2	6.9	7.1	4.9	4.1	2.6	2.4	0.0
New cases^b^	14	16	18	12	13	13	7	10	11	6	5	2	0	0
Cow-years at risk^c^		239		293		284		272		276		280		198
Apparent incidence^d^		0.13		0.10		0.09		0.06		0.06		0.02		0
**Herd B**														
Total positive cows	9	8	6	3	6	5	4	3	3	2	5	5	1	0
Animals tested	106	122	128	128	113	113	115	114	111	109	113	109	82	1
Apparent prevalence	8.5	6.6	4.7	2.3	5.3	4.4	3.5	2.6	2.7	1.8	4.4	4.6	1.2	0.0
New cases	9	1	2	0	5	4	1	0	1	1	4	1	0	0
Cow-years at risk		72		99		95		94		93		83		37
Apparent incidence		0.14		0.02		0.10		0.01		0.02		0.06		0
**Herd C**														
Total positive cows	0	17	26	23	19	22	18	20	18	15	13	8	7	0
Animals tested	0	121	145	149	178	161	145	155	157	145	142	117	102	0
Apparent prevalence	NA	14.0	17.9	15.4	10.7	13.7	12.4	12.9	11.5	10.3	9.2	6.8	6.9	NA
New cases	0	17	9	7	5	9	5	4	4	5	2	2	1	0
Cow-years at risk		13		114		123		110		117		108		33
Apparent incidence		1.27		0.14		0.11		0.08		0.08		0.04		0.03

^a^Test positive cows by considering enzyme-linked immunosorbent assay (ELISA) testing, fecal testing and tissue testing.

^b^Number of cows tested positive for the first time

^c^Observation time (in years) from entry in the study (at the first testing) until each cow tested positive or left the study (by culling, i.e. the infection status of the cow is right censored)

^d^New cases per year / cow-years at risk

**Fig 3:**
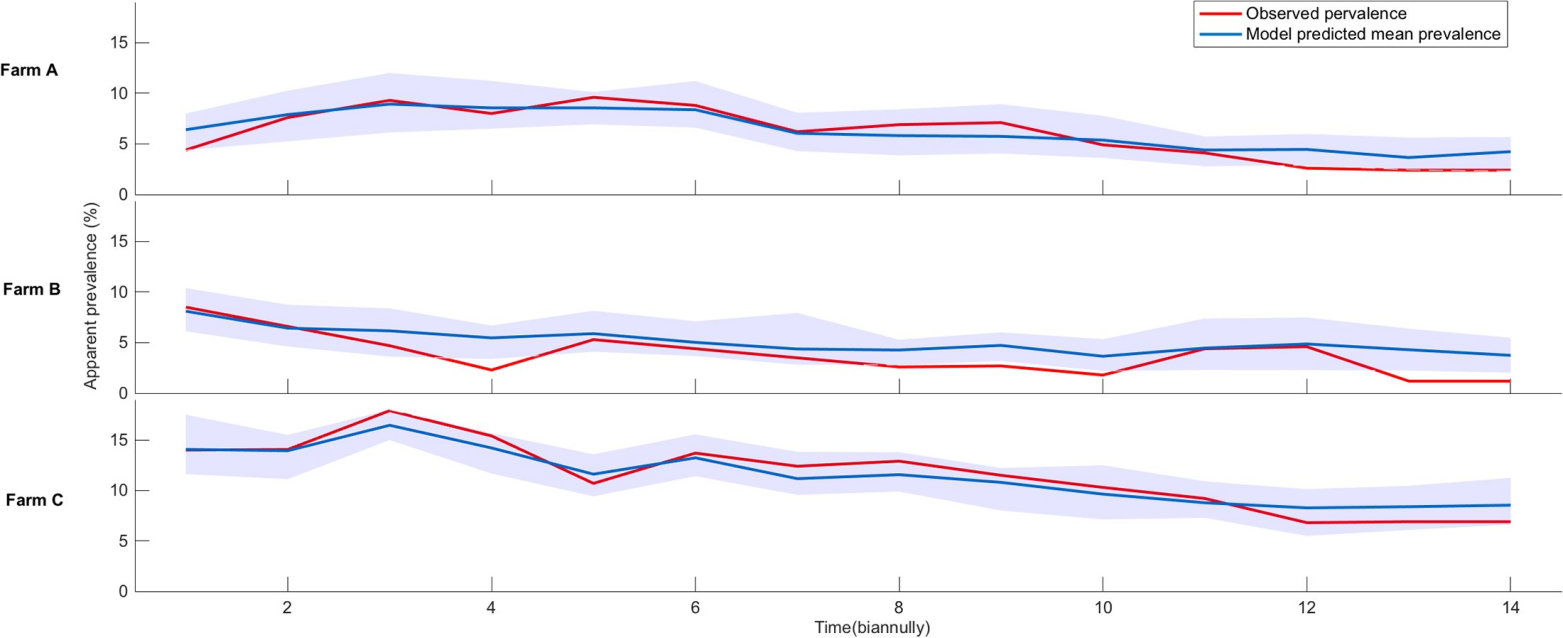
The fitting results of three *in silico* herds A (top), B (middle), and C (bottom) compared to the observed apparent prevalence for 7 years by biannual sampling. The shaded region shows the 95% confidence interval of the best 1% simulation runs.

Fig 3 shows the model predicted prevalence with a 95% confidence interval while fitting against the observed prevalence. It should be noted that our model confidence interval slightly overpredicts the prevalence of herd B, but for other two herds, it forecasts the best fitting. Through this model fitting exercise, our aim was to estimate the critical infection parameters for each herd, so that we can suggest herd specific intervention strategies.

### Estimated parameters

Table 5 provides the best fit estimates of herd-specific infection parameters for three northeastern dairy herds. Among the three herds, the model suggested that dam-to daughter transmission routes were the major transmission routes with the coefficient (*β*_*a*_) values of 0.4046, 0.1781 and 0.825 for farm A, B, and C, respectively. Environmental contamination (*β*_*environment*_) was the second major transmission routes while adult-to-adult transmission route was ranked third. Interestingly, we found that the importance of adult-to-calf transmission was highest in herd C, in which the initial number of latent animals were highest in numbers among the three herds. Based on the best 1% parameter sets, herd C again had the highest number of latent animals present (shown in supplementary S1 Table).

**Table 5:** The values of fitted parameters for three farms A, B, and C.

Parameters	Herd A	Herd B	Herd C
Adult to adult transmission coefficient (*β*_*A*_)	0.0069	0.0023	0.0005
Adult to calf transmission coefficient (*β*_*a*_)	0.40	0.18	0.86
Environmental transmission coefficient (*β*_*environment*_)	0.087	0.071	0.016
Calf to calf transmission coefficient (*β*_*c*_)	5.3×10^−06^	3.61×10^−06^	5.2×10^−06^
Heifer to heifer transmission coefficient (*β*_*h*_)	4.36×10^−06^	1.18×10^−06^	1.98×10^−06^
Initial Latent animals (*H*_*i*_)	18	12	81
Initial low shedding animals ($Y_{1_{i}}$)	15	2	12
Initial high shedding animals ${\;(Y}_{2_{i}})$	22	8	9

It is also noticeable that herd A has the highest adult-to-adult transmission probability among the three farms. Also, the initial starting distribution of the infected animals was very important for the fitting. It is seen that herd C start with the highest proportion of latent (73%) and low shedding (31%) animals among the three farms. The best-fitted parameters set is shown in the supplementary table (shown in supplementary S1 Table).

### Intervention strategies

**Fig 4:**
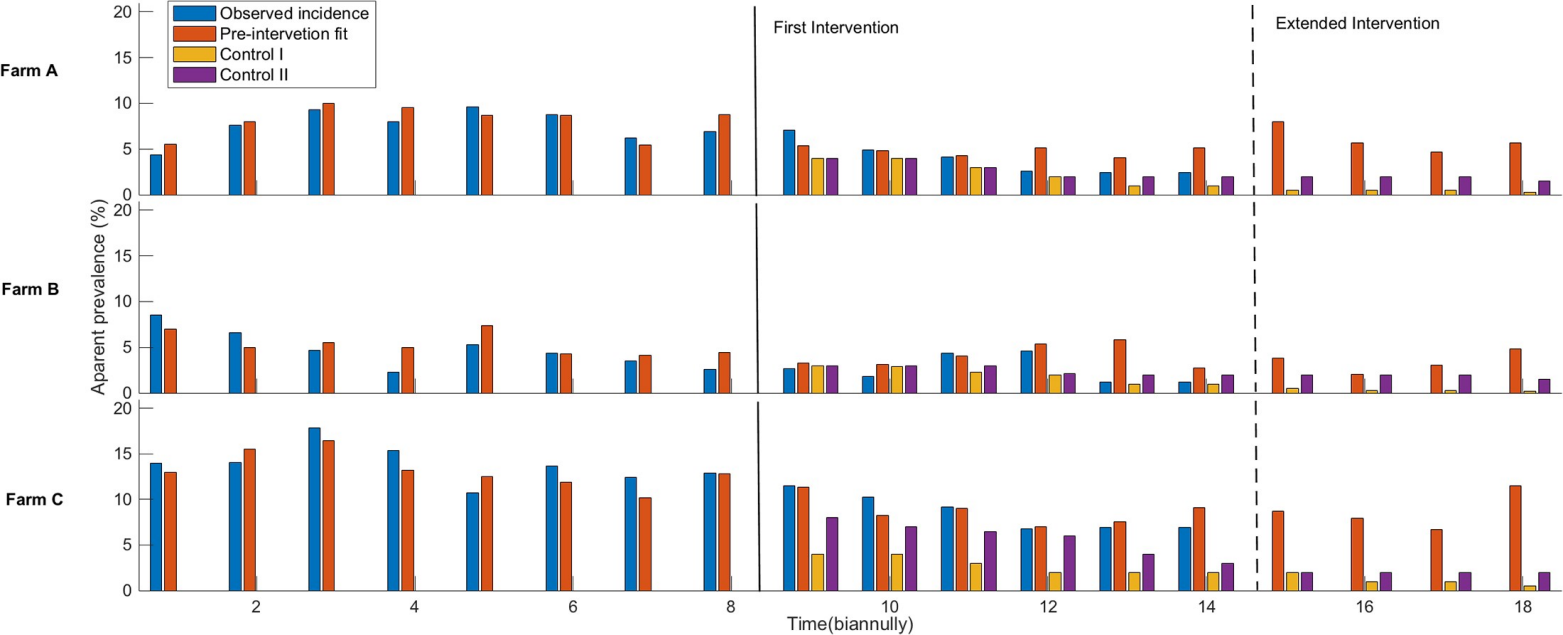
The apparent prevalence during the pre- and post-intervention period during the simulation of three *in silico* herds with two control strategies. Control I: culling red animals immediately and control II: culling only red animal with a delay of 305 days in milk. The two control measures are simulated in separating runs of the three *in silico* herds.

Once the three *in silico* herds were obtained from the fitting exercises, our next aim was to test the risk-based test and culling policy for each farm. Fig 4 presents the summary of the pre-intervention, post-intervention, and extended intervention results to the three fitted dairy herds. The results clearly show that the suggested intervention policy reduces the overall apparent prevalence for three herds, but it is noticeable that for high endemic herds the risk-based culling was comparatively less effective than the low endemic herds. To investigate further, we extended our intervention 2 years beyond the observations, but we did not see any elimination of MAP infection for the risk-based culling policy with control II. Culling red animals immediately (control I) was the best policy for all herds to decrease prevalence. Furthermore, we also calculated the number of years taken by the model to reduce the prevalence by 25% and 5% while two control programs were implemented after the pre-intervention period for three farms (shown in S2 Fig).

## Discussion and conclusion

Paratuberculosis is endemic in the bovine population in the US, which makes elimination unlikely at this time. When elimination is not possible, we have to rely on implementing the best herd-specific control strategies. Previous mathematical modeling studies show variable results for investigating infection dynamics [28,30,32], test-and-culling strategies [30,53], vaccination [29,54,55], and intermittent MAP shedding [35,47]. None of these combine the individual animal’s information with herd management policy while fitting the model to real herd data. To enhance this effort, IBM techniques can play an important role in modeling decisions for individual animal in an endemic herd. However, implementing the IBM results in real herds require realistic estimation of the herd and infection-specific parameters to test different intervention strategies. This paper presents an IBM framework of MAP where the model predicted infection dynamics are fit to and validated by the observed datasets conducted in three Northeastern US dairy herds. The fitting results show that the IBM is capable of reproducing the observed milk yield of each herd and estimating key herd-related parameters. Next, the model estimates critical transmission parameters for three herds and provides the best fit to the observed apparent prevalence data. Finally, we applied risk-based test and culling intervention strategies to the best fitted in silico herd models, showing that suggested interventions may be more beneficial for low prevalence herds than for moderate prevalence herds.

Precise information about the animal’s daily life events, such as age, milk yield, parity status, infection status, clinical signs, and adult, calf and heifer rearing management policies may assist to design real-world control strategies. Many MAP infected animals remain subclinically affected at the time of the initial infection, so precise information on the infection status of animals, combined with their economic status, is valuable for implementing effective and economically viable control strategies. The base herd fitting results suggest that the IBM recreates the observed animal distribution of the real herds (shown in Table 1). It also captures the observed milk yield curves and provides the estimates of herd-specific milk yield parameters. This feature is very important to evaluate the economic efficacy of the implemented interventions [56]. Lower milk yield is an important factor in the decision to cull an animal, but when culling an animal due to PTB test results is recommended, the effect on milk yield is not necessarily understood [57]. This leads to a lack of interest, on behalf of the herd manager, to cull based on test results alone. We observed this in the RDQMA herds, where only 0.01% of culling decisions were listed as being due to PTB, and all of those were in high-shedding animals. Furthermore, previous studies show that low- and high-path animals produce more milk before their first positive test than always-negative animals, especially high-path animals [14,35,58]. Mean milk production decreases after a first positive test, although non-progressing animals recover some productivity over time [57,58]. To account the overall impact of milk yield on culling, we used threshold values of milk yield for parity 1 and 2 for each farm and calculated median milk yield values for each parity from observed data.

The in silico dairy herd models reconfirm the apparent prevalence trends of the RDQMA herds (shown in Fig 3). In reality, calculation of the real-time prevalence is a complex process because such fine-grained testing detail is rarely available immediately. Moreover, different testing methods and variable test sensitivities make the task more difficult. Here, we used antemortem ELISA, fecal testing and postmortem tissue and fecal testing results to determine the test positive animals. For antemortem fecal culture tests, the sensitivity is determined to be 23–29% for infected cattle and 70–74% for infectious cattle [24]. At the slaughterhouse, the histology of biopsies yields sensitivity of 90 ± 5% and specificity of 100% and tissue culture yields sensitivity of 60% and specificity of 100% [59]. In comparison, ELISA tests used by the RDQMA study provide 20% sensitivity for infected cattle and 96% specificity; these numbers are aligned with the previous reports by Nielsen and Toft [60]. For simplicity, we chose a range of 25–35% sensitivity for infected animals and 96% specificity. It is one of the limitations of our model that we chose combined sensitivity and specificity estimates, whereas real herds may choose among three different testing strategies (fecal culture, ELISA, and PCR). With fecal culture, the results may take up to 3 months. Because of the time delay, many farmers rely on imperfect ELISA testing. Testing practices and recommendations vary in different geographical regions, with some finding that strategies like adaptive test scheme, age-specific sensitivities, and frequent testing can improve control. Recently, a simulation model built for the standard Danish dairy herd suggested an adaptive test scheme [8]. The age-specific test sensitivities were calculated from the test-records of 18,972 Danish dairy cows with MAP specific IgG antibodies on their final test-record [61]. However, care should be taken because using frequent testing may result in culling of many false positive animals [47].

To control PTB, it is important to determine which transmission routes are playing a major role in MAP transmission and persistence in the farm. Traditionally, the dam-to-daughter route is considered the primary route for transmitting MAP, but it can vary due to herd management policies (8). It is difficult to estimate this parameter directly from the epidemiological data due to imperfect testing and the wide variety of management policies. The role of environmental contamination is also difficult to measure from the epidemiological data, as MAP is pervasive within a dairy herd. However, our model suggests that dam-to-daughter transmission is indeed the primary transmission route, while environmental transmission is secondary route, in all three RDQMA herds (shown in Table 5). In our longitudinal data, we have the results of culturing environmental samples collected quarterly from several locations from farms. The culture results suggest that manure storage areas and shared alleyways were most likely to be positive for three herds [59], but no relationship was found between non-pen environmental sample status and the distance between shedding animals and the sample’s location, and neighboring pens did not significantly affect the results of the pen-level analysis. In our model, we included *β*_*environment*_ by using a probability distribution for the sake of simplicity. A recent mathematical study presented a theoretical method to quantify the level of environmental contamination through fecal-culture [62]. To quantify the precise role of different environments, further investigation of infection sources may be needed, potentially by examining the pathogens’ genomic sequencing data. Our model is adaptive in nature and can include a rigorous assessment of environmental contamination once data become available.

To date, the best-suggested control strategies against MAP are test and cull strategies. However, targeted test and cull requires combined information of individual animal, herd management, and hygiene policy. In a previous effort, we suggested risk-based culling strategies with four different options: aggressive culling, culling open red cows after 305 DIM, culling dam and offspring, and culling dam but not the offspring and we tested these intervention strategies along with different hygiene conditions on endemic herds [37]. In this study, we further investigated two risk-based control strategies: aggressive culling and culling open red cows after 305 DIM on three pre-fitted herds. We found that Control I resulted in the probability of PTB elimination being 0.24 after three years and 0.47 after five years in a low endemic herd (farm B). We also found that culling of open red cows after 305 DIM resulted in the probability of PTB elimination being 0.11 in three years and 0.24 after five years. Previous modeling studies also predict PTB elimination in low endemic herds [31,32,36,46,47]. In high endemic herds (such as farm A), however, we found the probability of PTB elimination to be only 0.06 after 5 years using Control I, and we did not find any elimination for farms C and A when using Control II over five years.

For moderate and higher endemic herds, farmers will likely want to focus on reducing prevalence, rather than elimination, and it is important to simulate how long it takes to reduce the prevalence to below a certain level. S2 Fig shows that a low endemic herd is likely to reach 5% of initial prevalence within two years for Control II, while a high endemic herd needs extended time to reach to that point, more than ten years in some cases. These suggest that culling high shedding animals may not provide elimination in high endemic herds, although it can lower the prevalence. Kirkby et al. showed that serial testing, along with hygiene, play a critical role in the PTB elimination process in Danish dairy herds, but these may not be economically justifiable [8,32,36]. These models were parameterized specifically for Danish conditions, so caution should be taken in transferring conclusions to other countries. Control activities are not uniformly coordinated nationally and internationally due to the variation in different farm management policies and government programs. It is important to note that our current model does not include any economic justification of the suggested control strategies, but the same base model has previously been used to show the economic justification of culling in case of the MAP in a separate study [56].

In conclusion, an important aspect of the model building is to validate the model with real-life data. In this study, we present an IBM framework of infectious disease in livestock operations and validate it using a longitudinal dataset from three northeastern dairy herds. The assessment of model predictions has lead us to the conclusion that the evaluation of modeling results is still a combination of intuitive model results, validation of the model with quality data, assumptions integrated into the modeling process, and estimation of key critical parameters. Moreover, this study opens multiple paths for further investigations. The extended model can include the impact of MAP infection on milk yield while including the economics of milk production for these three farms. Another extension of the model may include the clinical and molecular data of the infected animals, but adding molecular data requires further quantification of who infects whom (62,63). The current model is adaptive in nature, allowing us to add strain-specific data for each individual animal. This framework can be adapted for other infectious diseases to quantify the importance of key transmission routes and individual-level data to population-level phenomena, and to make decision based on implemented intervention policies. In summary, the quality of the conclusions drawn from model studies is closely linked to the quality of the data used for estimation of the parameters and model validation. Models validated with real-world data are more likely to produce useful and valid results.

## Supporting information

S1 Table The best 1% parameter sets were ranked from the parameter searching space. (DOCX)

S1 Fig The model predicted fitted to the observed milk yield for 360 days in milk for farm A, B, and C. The milk yield was calculated using equation shown in the method section.
(TIF)

S2 Fig The model predicted median number of years to reduce the apparent prevalence by 25% (top panel) and 5% (bottom panel) calculated from top 1% simulations with best set of parameters while implementing two control scheme I: Aggressive culling and control II: Delayed culling after the pre-intervention fit for the farms A, B, and C. (TIF)
