# Supplementary material for: A data-driven individual-based model of infectious disease in livestock operation: A validation study for paratuberculosis
Source: PLoS One. 2018 Dec 14;13(12):e0203177. doi: 10.1371/journal.pone.0203177 (PMC6294356; doi:10.1371/journal.pone.0203177)
Supplement: S1 Table — (DOCX) [file pone.0203177.s001.docx]

Table S1. The best 1% parameter sets were ranked from the parameter searching space.

| **Parameters** | **Herd A**  **Mean (95% CI)** | **Herd B**  **Mean (95% CI)** | **Herd C**  **Mean (95% CI)** |
| --- | --- | --- | --- |
| Adult to adult transmission coefficient $(\beta_{A}$) | **0.0033 (0.00062-0.0069)** | **0.0046 (0.0017-0.0075)** | **0.0041 (0.00047-0.0065)** |
| Adult to calf transmission coefficient ($\beta_{a})$ | 0.54 (0.11-0.96) | 0.37 (0.064-0.079) | 0.63 (0.055-0.9) |
| Environmental transmission coefficient ${(\beta}_{environment})$ | **0.053 (0.0089-0.090)** | **0.05 (0.0056-0.078)** | **0.046 (0.0036-0.087)** |
| Calf to calf transmission coefficient $(\beta_{c})$ | 0.69×10^-6^ (0.4×10^-6^-1.2×10^-5^) | 6.5×10^-6^ (0. 69×10^-6^-1.2×10^-5^) | 8.4×10^-6^ (0.18×10^-6^-1.2×10^-6^) |
| Heifer to heifer transmission coefficient ($\beta_{h}$) | 0.54×10^-6^ (0.53×10^-6^-0.1×10^-4^) | 4.9×10^-6^ (0.29×10^-6^-0.11×10^-5^) | 3.9×10^-6^ (0.35×10^-6^ -0.1×10^-4^) |
| Initial latent | 30 (3-75) | 10 (5-19) | 73 (52-85) |
| Initial low shedding animals | 18 (4-35) | 12 (2-36) | 31 (4-49) |
| Initial high shedding animals | 16 (7-23) | 11 (2-22) | 13 (2-23) |
