# Supplementary figures and images for: A data-driven individual-based model of infectious disease in livestock operation: A validation study for paratuberculosis
Source: PLoS One. 2018 Dec 14;13(12):e0203177. doi: 10.1371/journal.pone.0203177 (PMC6294356; doi:10.1371/journal.pone.0203177)

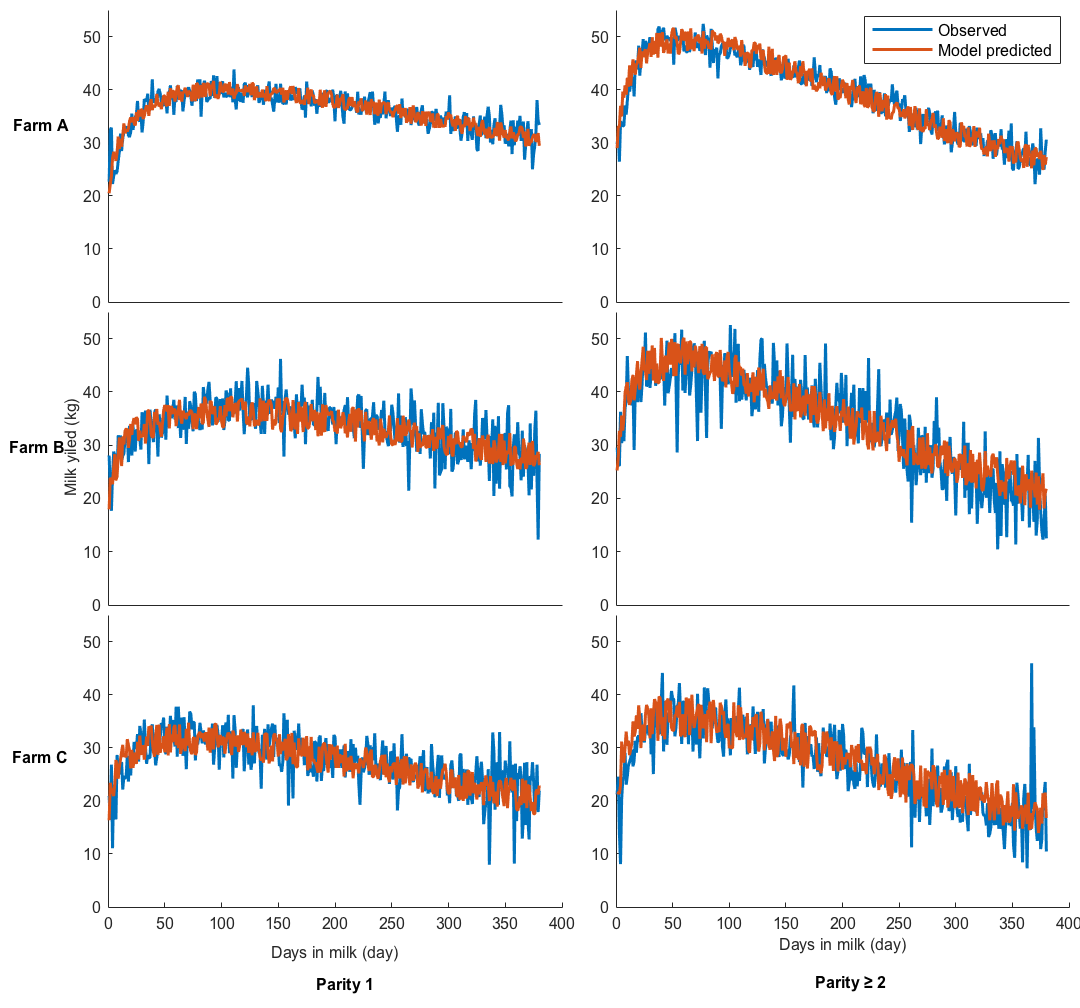

Supplement: S1 Fig — The milk yield was calculated using equation shown in the method section. (TIF) [file pone.0203177.s002.tif]

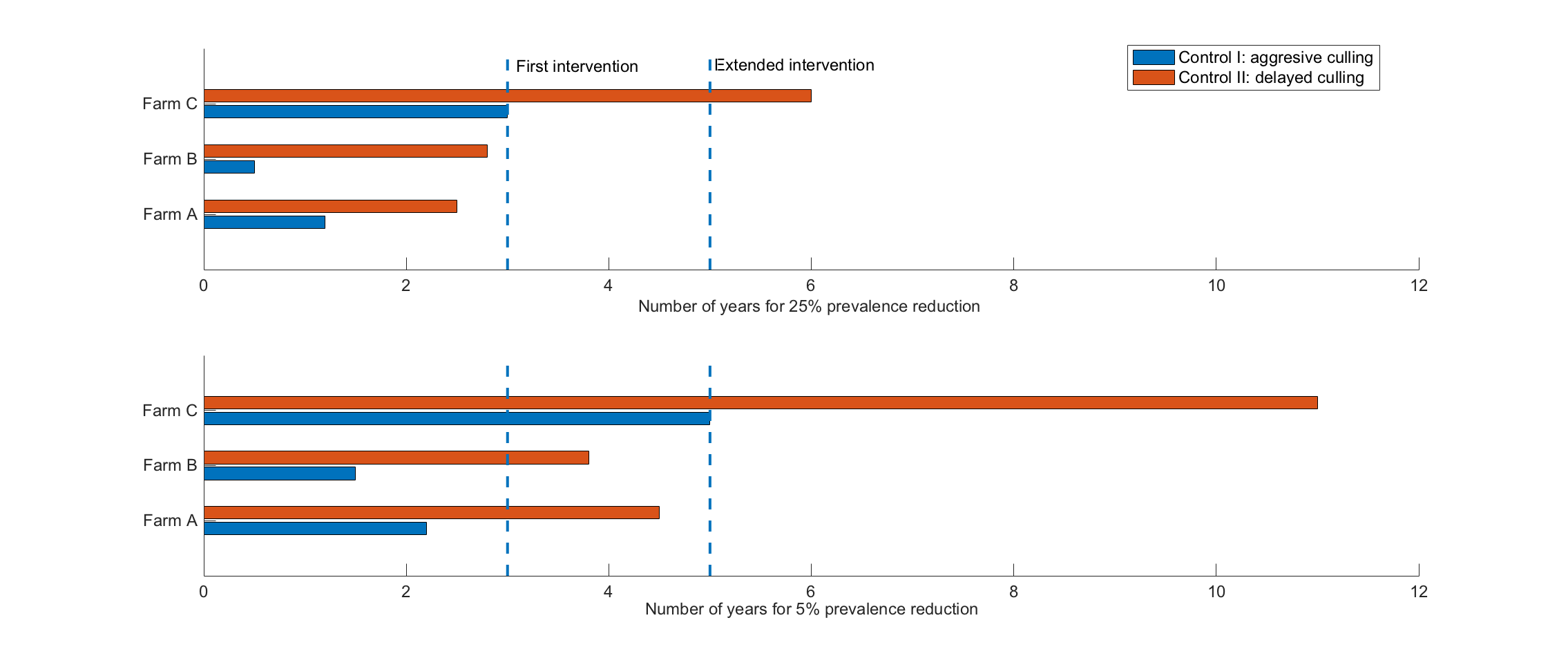

Supplement: S2 Fig — (TIF) [file pone.0203177.s003.tif]
